# Speckle Vibrometry for Contactless Instantaneous Heart Rate and Respiration Rate Monitoring on Mechanically Ventilated Patients

**DOI:** 10.3390/s24196374

**Published:** 2024-10-01

**Authors:** Shuhao Que, Iris Cramer, Lukas Dekker, Sebastiaan Overeem, Arthur Bouwman, Svitlana Zinger, Sander Stuijk, Fokke van Meulen

**Affiliations:** 1 Electrical Engineering Department, Eindhoven University of Technology, 5612 AZ Eindhoven, The Netherlands; s.que@tue.nl (S.Q.); i.c.cramer@tue.nl (I.C.); lukas.dekker@catharinaziekenhuis.nl (L.D.); s.overeem@tue.nl (S.O.); arthur.bouwman@catharinaziekenhuis.nl (A.B.); s.zinger@tue.nl (S.Z.); s.stuijk@tue.nl (S.S.); 2 Catharina Ziekenhuis, 5623 EJ Eindhoven, The Netherlands; 3 Kempenhaeghe, 5591 VE Heeze, The Netherlands

**Keywords:** camera, contactless, vital sign monitoring, laser speckle, speckle vibrometry, instantaneous heart rate, instantaneous respiration rate, ICU

## Abstract

*Objective*: Contactless monitoring of instantaneous heart rate and respiration rate has a significant clinical relevance. This work aims to use Speckle Vibrometry (i.e., based on the secondary laser speckle effect) to contactlessly measure these two vital signs in an intensive care unit. *Methods*: In this work, we propose an algorithm for the estimation of instantaneous heart rate and respiration rate from mechanically ventilated patients. The algorithm uses multiple regions, principal component analysis, and dominant angle analysis. A semi-automated peak detection method is implemented to precisely label the aortic valve opening peak within the cardiac waveform. *Results*: Compared with electrocardiography, the present work achieves limits of agreement of [−2.19, 1.73] beats per minute of instantaneous heart rate. The measurement spot is on the chest covered with two to three layers of duvet blankets. Compared with the airway flow signal measured by the mechanical ventilator, the present work achieves limits of agreement of [−0.68, 0.46] respirations per minute of instantaneous respiration rate. *Conclusions*: These results showcased Speckle Vibrometry’s potential in vital sign monitoring in a clinical setting. *Significance*: This is the first human clinical study for Speckle Vibrometry.

## 1. Introduction

Both heart rate and respiration rate are two vital signs that are routinely monitored in clinical settings. Continuous monitoring provides detailed information that can be used to assess the potential clinical deterioration of their patients, as both heart rate and respiration rate serve as critical indicators for the general health status of a patient [1,2]. The current gold standard for heart rate monitoring in the intensive care unit (ICU) is the electrocardiogram (ECG), which requires at least three electrodes to be attached to the patient’s skin. However, using wired sensors for heart rate monitoring is not always possible or desired due to a patient’s medical condition, such as severely burnt skin or delirium. Respiration rate monitoring is frequently performed using impedance-based measurement, whereas the gold standard is considered to be capnography [3]. These methods are obtrusive as well and are not always possible to be used in the general ward or ICU setting. Therefore, research efforts have been put into investigating using contactless solutions to monitor these two vital signs.

Several technologies have been proposed in the existing literature for contactless instantaneous heart rate and respiration rate monitoring. Methods for instantaneous heart rate monitoring are the use of cameras [4,5,6,7,8], RadioFrequency (RF) [9,10,11], and ultrasound [12]. Example methods for instantaneous respiration rate monitoring include the aforementioned three and thermography [13,14,15]. An RGB camera detects blood volume changes via visible skin and has been employed in both laboratory and clinical environments. Compared with sensors that detect micro-motions, including RF and ultrasound, it requires visible skin to detect heart rate. The RGB camera has also been used to extract respiration rate from the thoracic area which does not require skin exposure [8]. However, it requires sufficient ambient light whereas thermography can operate in no-light conditions to detect respiration rate. Both RF and ultrasound exploit the Doppler effect to detect minute displacements from the human body (e.g., the neck, the head, etc.) to derive cardiac and respiratory motions. Toften et al. [11] validated a radar-based sleep monitor to measure instantaneous respiration rate and compared its performance with respiratory inductance plethysmography. However, apart from the difficulty in separating cardiac motions from respiratory ones, RF and ultrasound have a limited measuring distance to reduce the impact of background noise (e.g., moving objects in the background, temperature, etc.).

Speckle Vibrometry (SV) is another contactless solution for the monitoring of instantaneous heart rate and respiration rate. An SV setup typically consists of a laser source and a camera that is defocused at the laser spot. It differs from laser speckle plethysmography (SPG) which requires visible skin and a focused camera. SPG extracts cardiac pulse signals based on certain statistical properties (e.g., mean intensity) of the laser speckles formed on the human finger [16]. While both SV and SPG exploit the secondary laser speckle effect [17], SV uses a defocused camera to capture a laser speckle pattern. Besides, SV employs motion registration techniques instead of statistical properties to extract micro-motions on the laser-illuminated surface. The advantages of SV over the aforementioned methods are its insensitivity to background noise and independence of visible skin. The feasibility of using SV to monitor average heart rate [18,19,20], instantaneous heart rate [21,22], and average respiration rate [19] has been described in existing literature, including our previous work [18,21]. However, there has been an absence of clinical studies on the implementation of SV in a real-life clinical setting.

In this work, we study the performance of using SV to monitor instantaneous heart rate in an ICU setting. We demonstrate the possibility of extracting respiratory motion modulated in SV cardiac motion signal, from which an instantaneous respiration rate can be extracted. Our previous work used SV for instantaneous heart rate monitoring in a controlled laboratory environment. In contrast, in this work we propose several new algorithms to address the practical challenges present in a real-life ICU setting. This is a special corner case where patients were sedated and mechanically ventilated after large surgery as well as the first human clinical application for SV.

## 2. Materials and Methods

### 2.1. Protocol

This study was part of the FORSEE study (NCT05455775), a prospective single-center validation study of video-based vital sign monitoring in ICU patients in a tertiary care hospital (Catharina Ziekenhuis, Eindhoven, The Netherlands). According to the Dutch directives on research in human subjects, the study was reviewed by the Medical Research Ethics Committees United (MEC-U) (Nieuwegein, The Netherlands, File no: W20.180). The study protocol was approved by the internal review board of Catharina Hospital Eindhoven on 9 March 2022. Written informed consent was obtained prior to the research procedure. All patients were mechanically ventilated after cardiac surgeries. Depending on the clinical situation, patients were either sedated with additional propofol administered or in the process of waking up. For practical reasons, we collected 20 min of measurements from SV and reference setup simultaneously from each patient.

### 2.2. Measurement Setup and Data

**Figure 1:**
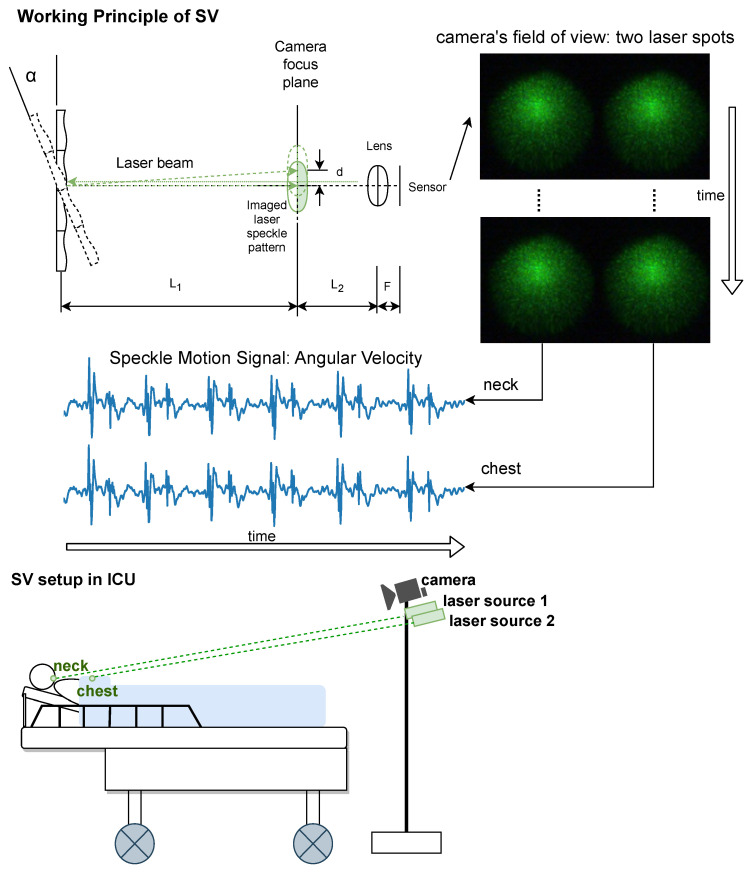
The upper plot illustrates the working principle of SV with two laser spots in one camera’s field of view, where $L_{1}$ denotes the distance between the focal plane of the optical system and the measured surface (i.e., neck and chest), $L_{2}$ denotes the distance between the focal plane of the optical system and the camera lens, *F* denotes the focal length of the camera lens, α denotes the angular change of the measured surface, and *d* denotes the displacement between laser speckle patterns of consecutive frames. The lower plot illustrates the SV setup in the ICU, where the two laser sources are mounted close to each other and parallel to the camera. Both laser sources share the same $L_{2}$ and *F* values, which are 0.4 m and 0.05 m, respectively. The $L_{1}$ of the laser source 1 is around 1 m while that of the laser source 2 is around 0.9 m.

A typical SV setup consists of one laser source and a camera that is defocused at the laser-illuminated spot. In the camera’s defocused field of view, a random intensity pattern of bright and dark spots can be observed. When the illuminated surface is optically rough on the scale of the laser wavelength, the reflected laser light waves differ in the path lengths. And that leads to constructive and destructive interference. This is due to the monochromatic and phase-coherent nature of the laser light. The SV setup, as used inside the ICU, is shown in Figure 1. It comprises a near-focused camera (i.e., the focal plane is between the detected surface and the camera lens) and two laser sources. The relative positioning of the camera and laser sources is fixed during recording, rendering the location of the two laser spots inside the camera’s field of view also fixed. Two regions of interest containing the two laser spots are manually selected. The two laser sources are placed next to each other and parallel to the camera. One laser source is aimed at the neck of the patient and the other is aimed at the thoracic area (i.e., on blankets). The thoracic area was the main focus of our investigation on instantaneous heart rate and respiration rate monitoring in an ICU setting. However, we also included the neck because it is relatively distant from the heart and has weaker cardiac motions. Thus it was considered to be a challenging spot and served as SV’s technical validation. Under the given imaging condition, angular changes of the detected surface are magnified significantly whereas transversal or axial movement barely has an impact [23]. The equation that maps the displacement between consecutive laser speckle patterns to the angular change of the detected surface is detailed in our previous work [18].

A monochrome camera with a 2.35 megapixel CMOS sensor (UI-3060CP-M-GL, IDS Imaging Development Systems GmbH, Dimbacher Str. 10, 74182, Obersulm, Germany) was used. Under a two-area setting, the camera operated with a field of view consisting of 1000-by-500 pixels (i.e., 500-by-500 pixels per area). It recorded 300 frames per second (fps) with an exposure time of 3.000 ms. Two class-I green lasers (Starlight Lasers X1 Groene Laserpen, Laserpenonline.nl, Kagerplein 415, 2172 EG, Sassenheim, The Netherlands) were mounted in parallel with a C-mount camera lens (M111FM50, Tamron, Saitama, Japan). Both laser light sources have a wavelength of 532 nm and an emission power of <1 mW. The camera lens’ aperture (f-stop) was set at F/1.8.

As per standard of care, heart and respiration rate reference data were provided using an IntelliVue MX750 patient monitor (Philips, Eindhoven, The Netherlands) and extracted using ixTrend (ixitos, Aken, Germany). Heart rate was derived from a single-lead ECG II (500 Hz) and respiration rate was derived using the airway flow (AWF) signal (125 Hz) from the mechanical ventilator (Hamilton Medical, Bonaduz, Switzerland). Measurements from the patient monitor and SV measurements were synchronized with timestamps at a precision of 1 ms.

### 2.3. Cardiac Motion Extraction

**Figure 2:**
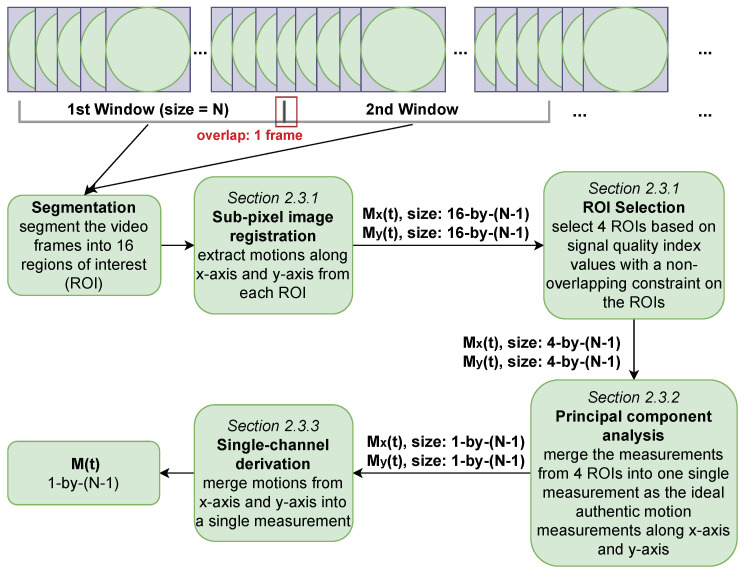
Summarized workflow of our novel motion (i.e., displacements between consecutive video frames) extraction algorithm (MRPD). The sliding window size is 10 s with an overlap of one video frame. The first window has a length N equal to 3000 video frames (i.e., 300 fps) while the following ones have a length N equal to 3001 video frames, with each window starting from its previous window’s last video frame.

For the extraction of cardiac motion, which manifests as angular velocity on the laser-illuminated surface, we propose a Multi-ROI Principal-component-analyse and Dominant-angle-analysis method (MRPD). The MRPD method is based on the existing full-frame cross-correlation method (FFCC) [18,19,21,24]. The workflow of our MRPD method is presented in Figure 2 and consists of three main parts, which are applied to each 10-s window of video recordings.

#### 2.3.1. Sub-Pixel Image Registration with ROI Selection

**Figure 3:**
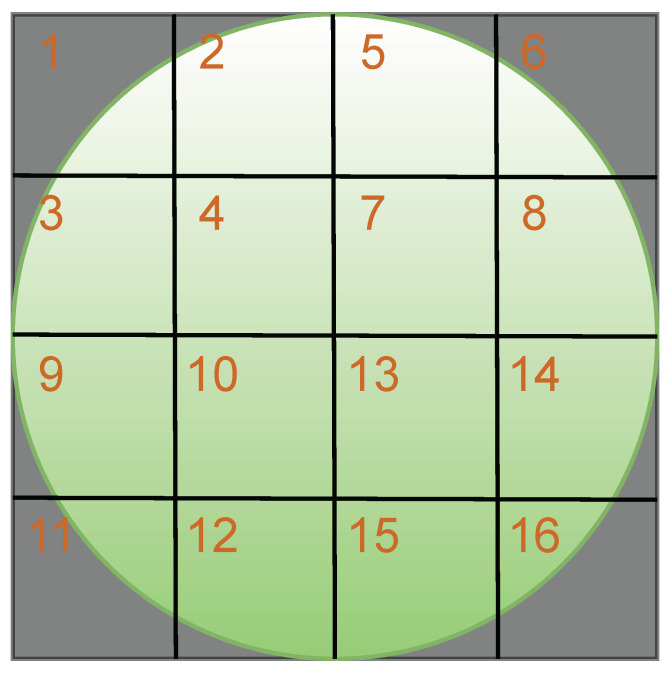
Regions of interest (ROI) indication and annotation for a full video frame of the laser speckle pattern (the green circle) in a dark background. The numbers indicate the index of each ROI.

Instead of extracting angular velocity from the whole video frame of the laser speckle pattern, each video frame was segmented into 16 ROIs equal in size as shown in Figure 3. The size of each ROI is 125-by-125 pixels. This ROI size was chosen based on the following reasons. First, it covered a sufficient area to avoid the de-correlation between consecutive video frames and thereby guaranteed the ability to extract movements of large magnitude. Second, if the ROI size was bigger (e.g., 250-by-250 pixels), the noise component could not be canceled sufficiently due to the limited number of ROIs available (i.e., 4 in this case). If the ROI size was smaller, the signals extracted from each ROI could be noisy, and more computational power would be needed. Last, it was square instead of rectangular or circular because it was not known beforehand in which direction the laser speckles moved between frames. To extract angular velocity signals from each ROI, i.e., displacements between consecutive video frames, we utilized the sub-pixel image registration algorithm proposed by Guizar-Sicairos et al. [25] with a sub-pixel accuracy of 0.01 pixels. Per the 10-s window, for each ROI(i) with i indicating the index number of the ROI, the angular velocity signals along the x-direction and y-direction were estimated as $M_{x_{i}}\left(t\right)$ and $M_{y_{i}}\left(t\right)$, respectively.

It was assumed that the ROIs with the higher SNR values should be those that had the widest coverages of the green area, that is where the laser speckles are located. Hypothetically speaking, these ROIs should be 4, 7, 10, and 13. However, since the SNR distribution across the laser speckle pattern could be quite uneven due to, for instance, the detected surface’s texture and the ambient lighting condition, the SNR of the ROIs at the upper left corner (e.g., 4) would not be necessarily comparable with that of those at the lower right corner (e.g., 13). Therefore, we propose two signal quality index values to automate the selection of the best four ROIs. We defined them as heart rate power ratio and heart rate spectral entropy [26], denoted as $SNR_{HR}$ and $SE_{HR}$, respectively.

The heart rate power ratio is defined as:

(1) $$SNR_{HR}=\frac{PSD(0.5-3\mathrm{Hz})}{PSD(all)}$$

where PSD denotes the power spectral density. A range from 0.5 to 3 Hz was chosen, corresponding to a heart rate of 30 up to 180 beats per minute (bpm).

The heart rate spectral entropy is defined as:

(2) $$SE_{HR}=-\frac{\sum_{m}P\left(m\right)*\log_{2}P\left(m\right)}{\log_{2}N_{F}}$$

where $N_{F}$ is the total frequency points between 0.5 and 6 Hz with a frequency resolution equal to 0.05 Hz and P(m) denotes the probability distribution of the power spectral density. A range from 0.5 to 6 Hz was empirically chosen as the base heart rate range and its first harmonic for the ICU patient group. A lower $SE_{HR}$ value is desirable as it indicates clearer periodicities in the frequency domain, leading to a cleaner waveform in the time domain.

First, $SNR_{HR}$ was utilized to discard four ROIs where the heart rate frequency components of the angular velocity signals, i.e., $M_{x_{i}}\left(t\right)$ or $M_{y_{i}}\left(t\right)$, had the lowest power in the frequency domain (e.g., ROI 1, 6, 11, and 16). Then, from the remaining 12 ROIs, $SE_{HR}$ was utilized to select the best four ROIs with the lowest spectral entropy values (i.e., $SE_{HR}$). This procedure was performed on $M_{x_{i}}\left(t\right)$ and $M_{y_{i}}\left(t\right)$ separately.

#### 2.3.2. Principal Component Analysis

After the $M_{x_{i}}\left(t\right)$ and $M_{y_{i}}\left(t\right)$ of the selected four ROIs were obtained, they were mapped onto the direction along which the variance of the signal was the largest, respectively. It was assumed that the angular velocity signal was composed of two independent components, the authentic angular velocity component and the sensor noise component. The authentic angular velocity component consists of angular velocities induced by cardiac, respiratory, and arbitrary movements (e.g., motion distortions introduced by nursing staff). The angular velocity signals from ROI(i) were then further defined as follows:

(3) $$\begin{array}{c} M_{x_{i}}\left(t\right)=A_{x_{i}}\left(t\right)+N_{x_{i}}\left(t\right) \end{array}$$

(4) $$\begin{array}{c} M_{y_{i}}\left(t\right)=A_{y_{i}}\left(t\right)+N_{y_{i}}\left(t\right) \end{array}$$

where $A_{x_{i}}\left(t\right)$ and $N_{x_{i}}\left(t\right)$ denote the authentic angular velocity and sensor noise components along the x-direction. $A_{y_{i}}\left(t\right)$ and $N_{y_{i}}\left(t\right)$ denote the components along the y-direction.

It was assumed that authentic angular velocity components were correlated among different ROIs whereas the sensor noise component was uncorrelated. Based on this assumption, a cross-correlation matrix was calculated from the four-ROI set of $M_{x_{i}}\left(t\right)$ and another one from $M_{y_{i}}\left(t\right)$. From each of the two matrices, the eigenvector corresponding to the highest eigenvalue was derived using principal component analysis (PCA) [27]. Using the eigenvector as the weighting matrix, the four angular velocity signals per direction (x or y) were combined into one single merged measurement. The merged measurements are denoted, respectively, as $M_{x}\left(t\right)$ and $M_{y}\left(t\right)$.

#### 2.3.3. Single-Channel Derivation

The dominant-angle (DA) method, as presented in our previous work [18] was used to merge both angular velocity signals, $M_{x}\left(t\right)$ and $M_{y}\left(t\right)$, into a single-channel SV measurement M(t) that is independent of the camera-surface orientation.

### 2.4. Respiratory Motion Extraction

From the cardiac motion represented by the angular velocity and denoted as M(t), the respiratory motion R(t) was estimated. First, M(t) was numerically integrated and a fourth-order low-pass Butterworth filter was applied, with a cut-off value equal to 0.7 Hz (corresponding to 42 respirations per minute (rpm)). In this case, the respiratory motion is represented by the angular displacement. This method will be compared with the methods described in literature [28,29] of directly applying the same low-pass filter on M(t) to create the respiration signal $M_{L}\left(t\right)$.

### 2.5. Peak Detection

#### 2.5.1. The Cardiac Signals

**Figure 4:**
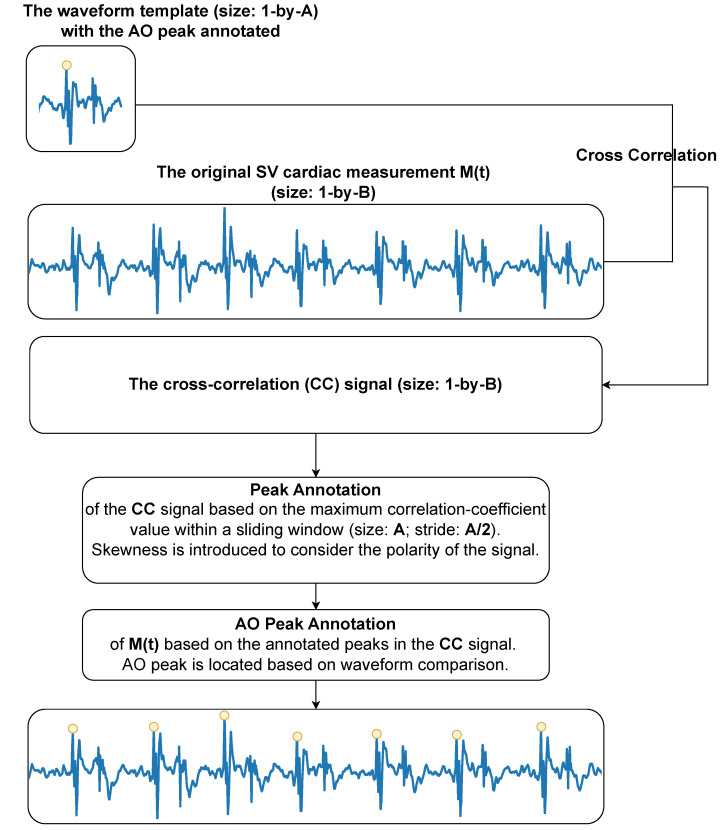
Summarized workflow of the waveform-based peak detection algorithm for the SV cardiac motion signal. The letter A denotes the length of the waveform template. The letter B denotes the length of the SV cardiac measurement M(t). CC denotes cross-correlation. AO denotes aortic valve opening and is annotated by a yellow circle in the blue line plot.

Similar to Gyrocardiography [30] which detects angular velocities on the chest introduced by the contractions of the heart, there is inter- and intra-subject variability observed in the SV waveform [18]. Different measurement spots (i.e., the neck and the chest) might yield different cardiac waveforms within the same subject. It was further observed that due to the intra-subject variability of the SV waveform over time, the peak of the highest amplitude within one cardiac cycle does not always correspond to the AO peak (i.e., the characteristic cardiac peak that corresponds to the aortic valve opening [30]). Sometimes the polarity of the SV waveform is flipped during recording (i.e., the amplitude of the AO peak switches from positive to negative values), which can be attributed to the respiration-induced displacement of the laser spot on the detected surface (i.e., a blanket). To reduce the influence of inter- and intra-subject variability of the morphology of the SV signal, a new semi-automated waveform-based cardiac peak detection algorithm was developed. This method starts with manually selecting one complete cardiac cycle with the AO peak annotated as the waveform template for each unique 20-min measurement, which is followed by an automated process. The automated process consists of two major steps: (1) locating the peaks of the cross-correlation signal between the waveform template and the whole SV cardiac measurement; (2) mapping these peaks to their corresponding AO peak locations based on waveform comparison. A workflow diagram is displayed in Figure 4. The details are presented in Algorithm A1 in the Appendix A.

Each cardiac cycle of an ECG signal is characterized by the QRS complex [31], among which the R peak (i.e., the highest amplitude in the R wave) is typically used for heart rate calculation. For the detection of R peaks in the reference signal, i.e., ECG, a widely adopted method that is a combined work of both W. Engelse and C. Zeelenberg [32] and A. Lourenco et al. [33] was used.

#### 2.5.2. The Respiration Signals

An adapted version of our previously developed algorithm was used for the estimation of instantaneous respiration rate [18]. An envelope signal was calculated, respectively, from $M_{L}\left(t\right)$, R(t), and the reference signal (AWF signal), by applying a fast Fourier transform-based bandpass filter (cutoffs: 0.1 Hz to 0.7 Hz). The envelope signal was then segmented into 20-s sliding windows with a 5-s stride. Within each 20-s window, the envelope peaks were labeled based on adaptive thresholding.

### 2.6. Instantaneous Heart Rate and Respiration Rate Estimation from Peak Alignment and Interval Selection

We employed the peak alignment and inter-peak interval selection algorithm proposed by our previous work [21] to align R peaks of ECG and AO peaks of M(t) and subsequently to select pairs of R-R peak intervals and AO-AO peak intervals. For each ground-truth R peak, within a given interval, a true AO peak was located. If there was more than one peak found within that interval of the SV signal, the peak that was most adjacent to the R peak was selected as the true AO peak (true positive peak), and the remaining peaks were discarded (false positive peaks). If there was no AO peak found within that interval, the R peak was discarded (false negative peak). After the one-to-one mapping between selected R peaks and AO peaks was obtained, inter-peak intervals were calculated from the consecutive R peaks and consecutive AO peaks, respectively. If between two selected R peaks, there had been originally one R peak but it was discarded during the peak aligning process, the interval formed by these two selected R peaks was labeled as “false intervals”. So were their corresponding AO-AO peak intervals. These inter-peak intervals were discarded to avoid overestimating the performance. The same method was used for peaks of AWF and peaks of R(t), and peaks of AWF and dips of $M_{L}\left(t\right)$. The peak of AWF denotes the maximum inspiratory flow rate measured at the end of inhalation. The peak of R(t) denotes the maximum angular displacement at the end of inhalation. The dip location of $M_{L}\left(t\right)$ corresponds to the peak location of R(t). After inter-peak interval selection, instantaneous heart rates [bpm] were calculated by dividing 60,000 milliseconds by each inter-peak interval [ms], and the same method was applied to calculate instantaneous respiration rates [rpm].

### 2.7. Evaluation Metrics

Bland–Altman analysis [34] was conducted to evaluate the performance of the estimated instantaneous heart rate and respiration rate from SV compared to their reference signals. The quantitative metric was the limits of agreement (LOA). A Bland–Altman analysis is valid only when the differences between measurements (i.e., ECG and M(t), AWF and R(t), and AWF and $M_{L}\left(t\right)$) have a normal distribution, which was verified by the Shapiro–Wilk test [35] with the *p*-value set to 0.05. The calculated limits of agreement were compared with the clinically acceptable LOA for instantaneous heart rate and respiration rate measurements according to the American National Standards Institute [36].

For inter-beat interval analysis, both the LOA and the root-mean-square error (RMSE) were used. The definition of the RMSE is given below:

(5) $$RMSE=\sqrt{\frac{1}{\left|N_{valid}\right|}\sum_{i=1}^{\left|N_{valid}\right|}{\left(IBI_{ECG}-IBI_{M(t)}\right)}^{2}}$$

where $IBI_{ECG}$ and $IBI_{M(t)}$ are selected pairs of R-R peak intervals and AO-AO peak intervals, and $\left|N_{valid}\right|$ denotes the total number of valid intervals.

## 3. Results

In total, 11 postoperative patients after cardiac surgeries were recorded with SV inside the ICU as a subset of the FORSEE study. Demographical information is presented in Table 1. From each patient, 20 min of data were recorded. For visualization clarity, a fixed set of colors is used to indicate the same patient in all graphs. One patient with a pacemaker was excluded from the analysis of instantaneous heart rate as no variation in heart rate was expected. The patient coded as purple does not have the chest measurement available due to a malfunction of the laser beam during recording.

### 3.1. Time Domain Analysis

**Figure 5:**
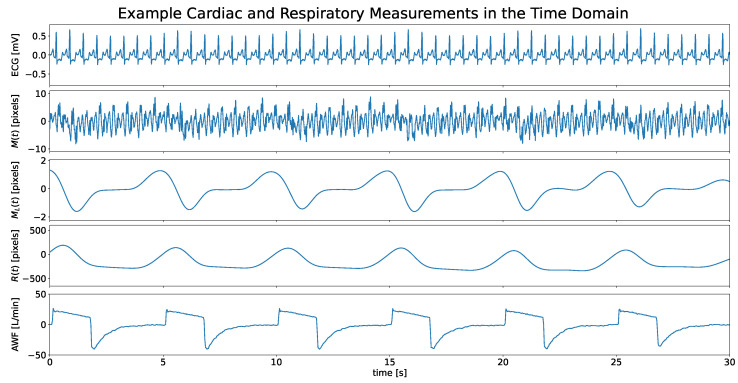
Example segments of cardiac and respiratory measurements in the time domain. M(t), $M_{L}\left(t\right)$, and R(t), respectively, denote SV cardiac motion, baseline wander of the SV cardiac motion, and SV respiratory motion signals. AWF is the respiration signal from the mechanical ventilator.

Figure 5 shows a segment of the measurements in the time domain from one patient, including ECG, M(t), $M_{L}\left(t\right)$, and R(t), and AWF. M(t) denotes the SV cardiac motion (angular velocity). It is observed that by extracting the baseline wander of M(t) a signal that has a respiratory periodicity can be obtained, denoted as $M_{L}\left(t\right)$. However, the pixel range of $M_{L}\left(t\right)$ only covers up to about four pixels, which is lower than the cardiac movements on the measured surface (about 20 pixels). This is because $M_{L}\left(t\right)$ only consists of respiration-induced low-frequency variations but not the respiratory motions themselves. In contrast, R(t) represents the cumulative pixel displacements (about 600 pixels and 1000 pixels, respectively), taking into account the co-effect of both cardiac and respiratory movements on the measured surface. R(t) also exhibits clear periodicity and can be used to extract instantaneous respiration rates. Of course, the pixel ranges here do not carry any absolute meaning. What differentiates R(t) from $M_{L}\left(t\right)$ is its waveform, which is a more authentic representation of the respiratory movement. Therefore, R(t) carries more information than $M_{L}\left(t\right)$, which entails not only the instantaneous respiration rate but also the respiratory motion. It is worth noting that the waveform of the AWF signal here indicates the airway flow, i.e., the volume of air passing through a point in the airway per minute. Therefore, although all three measurements exhibit respiratory periodicity, they are from different origins.

### 3.2. Inter-Beat Interval Analysis

Table 2 shows the performance of the inter-beat interval agreement (i.e., indicated by LOA ranges and RMSE values). When the laser spot was on the neck, the minimum mean RMSE value of 19.55 ms and LOA of [−37.68, 41.47] ms were achieved with the MRPD method combined with the waveform-based peak detection algorithm. Such improvement was significant, from 40.11 ms (achieved with the old method) to 19.55 ms. In contrast, when the laser spot was on the chest, the difference between the MRPD and FFCC methods was smaller, regardless of which peak detection algorithm was used. The minimum RMSE value of 11.90 ms and LOA of [−20.41, 25.32] ms were achieved on the chest. This relatively limited improvement (from 15.34 ms to 11.90 ms) on the chest could be attributed to the fact that the cardiac motions from the chest are easier to detect, rendering the signal quality sufficiently high for the FFCC and Hilbert methods to already yield adequate results.

Table 3 shows the impact of each component of the MRPD method on the performance, including the ROI selection, the number of the selected ROIs, and how the motion measurements from these ROIs were synthesized into one measurement. It can be observed that the best combination for both the neck and the chest is using both signal quality indices (SE and SNR) to select four ROIs and synthesizing them using PCA. This coincides with the results shown in Table 2. For the neck, both signal quality indices SE and SNR (i.e., which selected four ROIs) contributed to better results than when no ROI selection was performed (i.e., which used all 16 ROIs). The usage of PCA in general also yielded better results than the equal-weighted averaging method. As for the chest, it can be observed that the impact of PCA outweighed that of both SE and SNR.

### 3.3. Heart Rate Analysis

**Figure 6:**
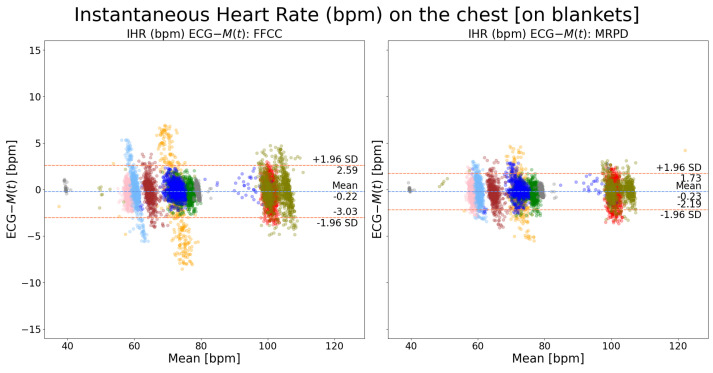
Bland–Altman plots of instantaneous heart rate on the chest (ECG-M(t)): a comparison between FFCC and MRPD motion extraction methods. Each color indicates recordings from one patient. The waveform-based cardiac peak detection algorithm was used. The FFCC method refers to the full-frame motion extraction algorithm while the MRPD method refers to our new algorithm: the multi-ROI-based motion extraction algorithm.

**Figure 7:**
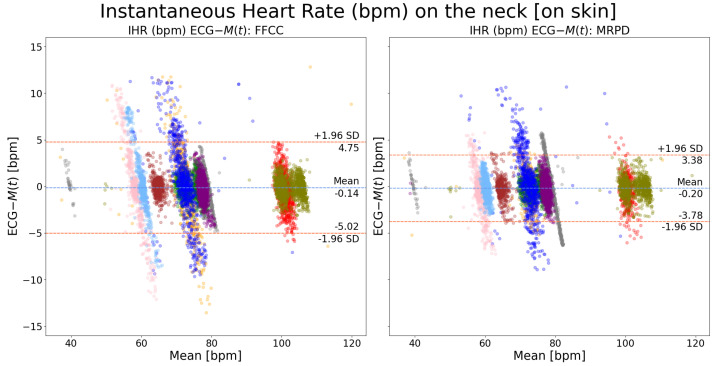
Bland–Altman plots of instantaneous heart rate on the neck (ECG-M(t)): a comparison between FFCC and MRPD motion extraction methods. Each color indicates recordings from one patient. The waveform-based cardiac peak detection algorithm was used. The FFCC method refers to the full-frame motion extraction algorithm while the MRPD method refers to our new algorithm: the multi-ROI-based motion extraction algorithm.

Figure 6 shows the performance comparison of measuring instantaneous heart rate on the chest between the adopted method in existing literature (FFCC) and our novel algorithm (MRPD). It can be observed that the LOA of MPRD outperforms that of FFCC by almost 1 bpm. Figure 7 shows a similar performance with SV measured on the neck. It shows that the LOA of MPRD precedes that of FFCC by slightly over 1 bpm. By comparing Figure 6 and Figure 7, we can observe that the performance on the chest exceeds that on the neck by around 2 bpm regardless of the motion extraction algorithm that was used.

**Figure 8:**
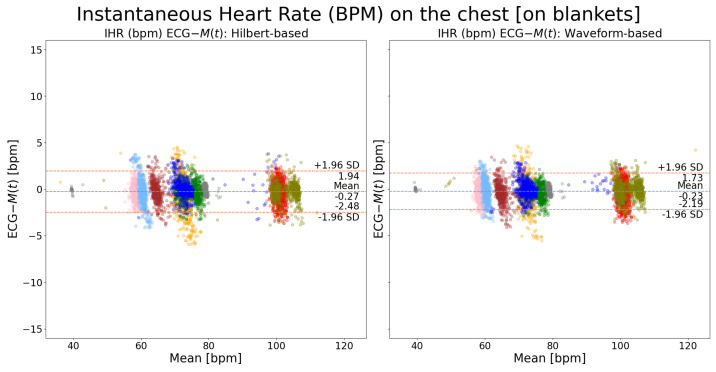
Bland–Altman plots of instantaneous heart rate on the chest (ECG-M(t)): a comparison between waveform-based and Hilbert transform-based peak detection methods. Each color indicates recordings from one patient. The MRPD motion extraction method was used.

**Figure 9:**
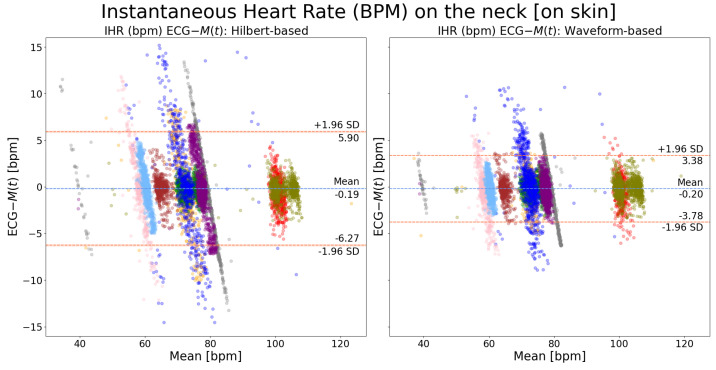
Bland–Altman plots of instantaneous heart rate on the neck (ECG-M(t)): a comparison between waveform-based and Hilbert transform-based peak detection methods. Each color indicates recordings from one patient. The MRPD motion extraction method was used.

To compare the performance of Hilbert transform-based and waveform-based peak detection algorithms, the MRPD method was utilized as the motion extraction method due to its superior performance showcased above. Figure 8 shows that there is no performance difference between these two peak detection algorithms when the measurement spot is on the chest. However, when the measurement spot is on the neck, as shown in Figure 9, the waveform-based algorithm outperforms the Hilbert transform-based algorithm by almost 2 bpm. Such an observation is consistent with the results shown in Figure 6 and Figure 7, where a larger improvement of MRPD over FFCC is found when the measurement spot is on the neck.

**Figure A1:**
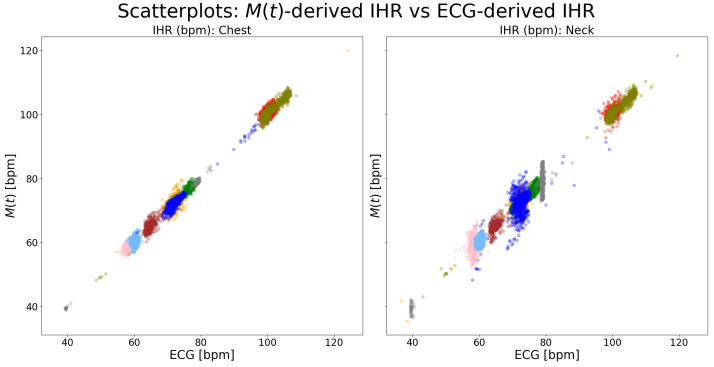
Scatterplots of instantaneous heart rates (IHR) derived from ECG and M(t). The MRPD method was used in combination with the waveform-based peak detection algorithm. Each color indicates recordings from one patient.

Several patients exhibited low heart rate variability (i.e., almost a vertical line in the Bland–Altman plot of the neck), such as the ones coded in pink, light blue, and grey (see Figure 6 and Figure 7). Right before the recording, these patients all went through aortic valve repair surgeries. During the recording, they were under the effect of propofol and noradrenaline. This could have potentially contributed to their low heart rate variability during the measurement. However, the grey patient had episodes of cardiac arrhythmia during measurement (i.e., variability in heart rate), which was accurately captured by SV. To observe better the heart rate variability of each patient, please refer to the scatterplots (see Figure A1) in the Appendix B.

No differences in performance were found between patients with additional propofol administered and those without. A detailed comparison is given in the Appendix C.

### 3.4. Respiration Rate Analysis on Mechanically Ventilated Patients

**Figure 10:**
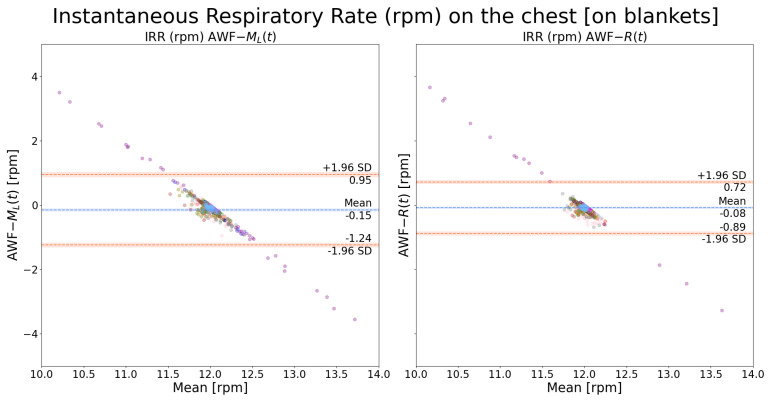
Bland–Altman plot of instantaneous respiration rate on the chest: a comparison between *AWF*-$M_{L}\left(t\right)$ and *AWF*-R(t). The AWF is the mechanical ventilation signal with a stationary frequency equal to 12 rpm. Each color indicates recordings from one patient.

**Figure 11:**
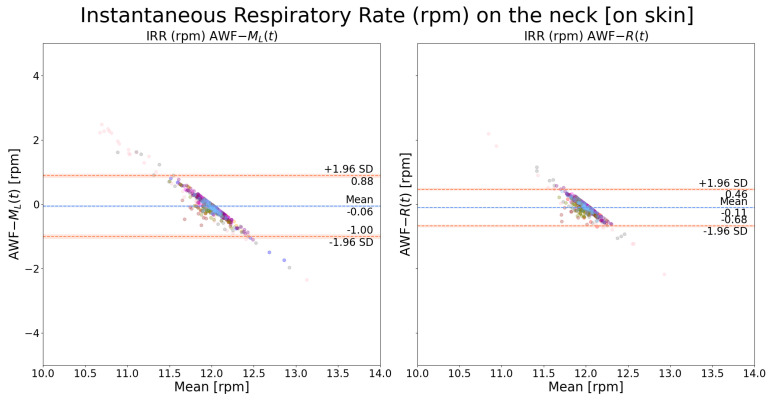
Bland–Altman plot of instantaneous respiration rate on the neck: a comparison between *AWF*-$M_{L}\left(t\right)$ and *AWF*-R(t). The AWF is the mechanical ventilation signal with a stationary frequency equal to 12 rpm. Each color indicates recordings from one patient.

Instantaneous respiration rates were extracted, respectively, from $M_{L}\left(t\right)$ and R(t) and the AWF signal was used as the reference. As shown in Figure 10, it can be observed that R(t) and $M_{L}\left(t\right)$ yielded very similar results when the measuring spot was on the chest, with the bounds of LOA around 1 rpm. In comparison, as shown in Figure 11, R(t) yielded slightly better results than $M_{L}\left(t\right)$. The points form a straight-line distribution because there is no respiration rate variability due to the mechanical ventilator.

## 4. Discussion

In this work, we have showcased the potential of SV in extracting cardiac and respiratory motions from mechanically ventilated patients in an ICU setting. SV is privacy-preserving by design because in a defocused camera’s field of view, the laser speckle pattern is the targeted area, and no visible human face can be discerned. During recording, the patients were all mechanically ventilated, and some were administered with additional propofol. It is a decent starting point for SV to be used in a real clinical environment with available high-quality and medically graded physiological measurements (e.g., ECG and AWF). The usage of a high-speed camera comes with an additional cost compared with, for example, simply using ECG electrodes. However, SV provides a contactless solution that is desirable and needed in certain clinical situations, such as in critical care on patients with burnt skin or delirium or sleep monitoring. In addition, the SV solution proposed in this work has a similar price range to other contactless sensing modalities.

There is inter- and intra-subject variability observed in the waveform of SV cardiac signal, M(t), due to the amount of biomechanical information it carries. The biomechanical information itself is subject- and time-dependent. This advantage also poses challenges in the accurate annotation of the characteristic peaks within one cardiac cycle (e.g., the AO peak). Our proposed waveform-based cardiac peak detection algorithm was designed and tailored to the specific needs of SV. It has achieved an improved result (as shown in Figure 8 and Figure 9) compared with the Hilbert-transform method. The latter annotates the peak reaching the highest amplitude within each cardiac cycle, disregarding the waveform variability over time.

When presented as inter-beat interval agreements, M(t) achieved an LOA of [−20.41, 25.32] ms on the chest and [−37.68, 41.47] ms on the neck. The performance of M(t) on the chest is comparable to the performance (an LOA of [−23.41, 25.45] ms) reported in [22] that used SV on the chest of healthy volunteers in a laboratory setting. Furthermore, it exceeds the performance (an SD of 25.2 ms) reported in [16] using SPG on healthy volunteers in a laboratory setting. When presented as instantaneous heart rate agreements, M(t) achieved clinically accepted results from the neck (an SD of 1.79 bpm) and the chest (an SD of 0.98 bpm). However, M(t) exhibits a performance discrepancy between the two measuring spots. We attribute such a difference to two plausible factors. First, the laser was directly illuminated on the surface of the skin when the measuring spot was on the neck whereas it was on two to three layers of duvet blankets when the measuring spot was on the chest. Considering the green visible light source (532 nm) that we deployed, the reflection was stronger on the duvet blankets than on bare skin, rendering a higher SNR of the laser speckle pattern formed on the former. Second, as discussed in our previous work [21], adding several layers of duvet blankets aided in the extraction of cardiac motions thanks to its dampening effect on low-frequency movements, such as respiratory ones. Respiratory movement has a higher magnitude than cardiac ones. Movements of higher magnitude cause the laser speckles to move at a larger distance in the camera’s field of view. When the frame rate is fixed (300 fps in our case), the larger the movement, the more difficult it is to extract the motion between consecutive frames, and the more noisy M(t) becomes. However, such a difference between these two measuring spots was significantly reduced (see Figure 8 and Figure 9), by approximately 2 bpm, when we applied the waveform-based peak detection algorithm (around 1.5 bpm) in place of the Hilbert transform-based method (around 3.5 bpm). This improvement by one fold solely based on the improvement of AO peak annotation indicates that the potential impact introduced by different measuring spots can be mitigated via algorithmic improvement. As shown in Figure 6 and Figure 7, the performance of SV is not dependent on heart rate values. Within our patient group, SV can measure from 40 bpm to 100 bpm without any performance bias. These two observations showcase the resolution of motions that SV can detect and its usability on real patients for instantaneous heart rate monitoring.

To evaluate how each component of our proposed MRPD method contributed to the performance, we present a comprehensive comparison in Table 3. It can be observed that when the PCA was used to synthesize motions from different ROIs, for the neck, a combination of both signal quality indices, SE and SNR, yielded better results than using either of them alone or none at all. However, for the chest, the aforementioned four cases yielded very similar results. When a simple equally-weighted averaging method was used in place of PCA, on the chest, the results were worse, especially when neither SE nor SNR was used to perform ROI selection. This could be attributed to the fact that the noise residing in different ROIs on the chest was uncorrelated (i.e., which can be detected by PCA) but did not exhibit characteristics that can be detected by either SE or SNR.

On the estimation of instantaneous respiration rate, both R(t) and $M_{L}\left(t\right)$ achieved clinically accepted results from both measuring spots in this corner case. The achieved LOAs are also comparable to the result ([−0.99, 0.85] rpm) reported in [11]. R(t), representing the respiratory movement derived from the numerical integration of M(t), yielded slightly better results than $M_{L}\left(t\right)$ which is the baseline wander of M(t). This demonstrates the potential of R(t) in the future as a contactless respiratory measurement that not only carries information on respiration rates but also movement. By aiming one laser spot on the chest and the other on the abdomen, R(t) extracted from both sites could perhaps be used to deduce respiratory efforts or detect desynchronized thoracic and abdominal movements that can occur during an apnea episode.

There are several limitations of this study. The current waveform-based method is semi-automated. More specifically, the determination of the waveform template from one complete cardiac cycle is manual. Future work needs to investigate automating this step (e.g., via ensemble average), which shall also introduce a more dynamic construction of waveforms that could self-adapt over time in case of waveform changes during a longer duration of recording (e.g., more than 20 min). The patient group is special in the sense that some of the patients were under the effect of additionally administered propofol. Second, the patients were mechanically ventilated and not breathing spontaneously. Although no definite or concrete claims have been made, studies have indicated the potential dampening effect of propofol on heart rate variability [37], which is a relevant factor to consider for instantaneous heart rate monitoring. Nevertheless, within this study, no difference can be observed between SV’s instantaneous heart rate performances in patients who received additional propofol and patients who did not. The number of patients in our experiment is small. Under the same monitoring conditions, our solution can be generalized to other situations as long as patients do not move frequently. However, as the number of patients increases, more variability of SV cardiac signal waveform can potentially occur. Its impact on the performance of our SV solution still needs further investigation.

Beyond the ICU setting, SV could have value in other clinical applications. For instance, in sleep monitoring, the patient does not move frequently and benefits from less obtrusive sensing techniques. In this case, SV could be a potential contactless addition or replacement for heart rate and respiration rate monitoring. Since SV detects respiratory motion, two laser beams aimed, respectively, at the chest and at the abdomen can be used to assess respiratory effort or potential desynchronization introduced by sleep apnea. In the general ward, where the vital signs are mainly measured based on spot-checks, SV could be a potential continuous solution to measure heart rate and respiration rate. However, when it comes to moving SV from this special corner case to a more general ward setting or sleep monitoring, there are several factors to be considered. First, we only recorded 20 min from each patient, which is too short of a window to detect potential deterioration of the patient. For long-term monitoring (e.g., 8–48 h), an alternative device setup entailing both hardware selection and software configuration might be needed. Second, we need to address potential motion distortions introduced by non-sedated patient’s spontaneous movement and tackle more diverse cardiac and respiratory patterns. In the context of long-term monitoring, such as a sleep study, occasional episodes of motion distortions caused by the patient’s spontaneous changing posture are often of limited duration. Thus, they could be simply removed from the analysis. It would not jeopardize the overall assessment of the patient’s health state during sleep. In the general ward, the patient is usually non-sedated and while in bed might be doing some reading or talking. In such cases, a potential solution could be to introduce several laser beams aimed at different spots of the body and bed. Through motion reconstruction from multiple sensory inputs, the cardiac motion could be potentially extracted from the original signal contaminated by distortions.

## 5. Conclusions

In this work, we demonstrate that SV can be used to extract cardiac and respiratory motions from real-world ICU patients through algorithmic improvements and innovations. Our multi-ROI-based method allows for the automated selection of high-quality speckle patterns for motion extraction and significantly reduces the impact of sensor noise. Our waveform-based method allows for precise AO peak annotation in the presence of diverse waveform patterns. The detailed motion waveform of SV allows the estimation of instantaneous heart rate and respiration rate as demonstrated by this work. It can also be used to potentially extract other cardiac and respiratory information, such as respiratory effort, sleep apnea, and cardiac arrhythmia, which deserves the attention of future work. This is the first real-world clinical study of SV as a contactless sensing technology that does not require visible skin. It also serves as a starting point to transition SV from a laboratory-controlled environment to a clinical one. Future work needs to assess the feasibility of using SV for longer recording duration, e.g., a minimum of 8 h in sleep monitoring or 48 h in a general ward setting, where patients are not sedated, breathe spontaneously, and can have spontaneous movements. Long-term monitoring on a larger population introduces higher variability of cardiac and respiratory motion patterns. The feasibility of using the current motion extraction algorithm for such diverse motion patterns still needs to be investigated. A fully automated accurate AO peak annotation method still needs to be developed accordingly.

## Figures and Tables

**Table 1:** Patient information. N = number of patients, IQR = interquartile range, BMI = body mass index. # denotes the number of.

Baseline Characteristics (N = 11)	
Age (median, IQR)	74, 15.5
BMI (median, IQR)	26.3, 2.4
Male	8
Female	3
#patients on propofol	7
#patients using pacemaker	1
Admission reason ICU	Postoperative after cardiac surgery

**Table 2:** Comparison of the inter-beat interval agreements. FFCC = full-frame based motion extraction method, MRPD = multiple-ROI based motion extraction method, Hilbert = Hilbert-transform based peak detection method, Waveform = waveform-based peak detection method, LOA = limits of agreement based on Bland–Altman analysis, RMSE = root-mean-square error, SD = standard deviation. The best combination of measurement spot and algorithms is highlighted in bold font.

Laser Spot	Motion Extraction	Peak Detection	LOA [ms]	RMSE [ms] Mean ± SD
neck	FFCC	Hilbert	[−89.47, 87.98]	40.11 ± 31.84
neck	FFCC	Waveform	[−67.90, 67.36]	31.74 ± 24.44
neck	MRPD	Hilbert	[−85.14, 83.11]	38.69 ± 21.69
**neck**	**MRPD**	**Waveform**	**[−37.68, 41.47]**	**19.55 ± 12.24**
chest	FFCC	Hilbert	[−29.18, 34.13]	15.34 ± 10.47
chest	FFCC	Waveform	[−30.38, 34.63]	15.78 ± 7.65
chest	MRPD	Hilbert	[−23.58, 29.25]	13.09 ± 6.55
**chest**	**MRPD**	**Waveform**	**[−20.41, 25.32]**	**11.90 ± 5.56**

**Table 3:** Comparison of the inter-beat interval agreements: the components of the multiple-ROI based motion extraction (MRPD) method. RMSE = root-mean-square error, SD = standard deviation. The best combination of measurement spot and MRPD components is highlighted in bold font. The motion synthesis method Average indicates averaging across all used ROIs instead of using principal component analysis. The waveform-based peak detection method was used.

Laser Spot	ROI Selection	Number of Used ROIs	Motion Synthesis of Used ROIs	LOA [ms]	RMSE [ms] Mean ± SD
neck	No	16	PCA	[−58.06, 60.59]	25.15 ± 20.36
neck	No	16	Average	[−74.61, 77.46]	30.57 ± 35.02
**neck**	**SE + SNR**	**4**	**PCA**	**[−37.68, 41.47]**	**19.55 ± 12.24**
neck	SE	4	PCA	[−42.95, 46.59]	20.94 ± 12.59
neck	SNR	4	PCA	[−51.62, 54.41]	23.82 ± 18.02
neck	SE + SNR	4	Average	[−42.78, 46.22]	20.90 ± 12.44
neck	SE	4	Average	[−55.50, 57.23]	26.28 ± 22.29
neck	SNR	4	Average	[−72.64, 72.82]	32.40 ± 32.41
chest	No	16	PCA	[−21.79, 21.17]	12.80 ± 5.76
chest	No	16	Average	[−43.77, 46.53]	20.15 ± 16.49
**chest**	**SE + SNR**	**4**	**PCA**	**[−20.41, 25.32]**	**11.90 ± 5.56**
chest	SE	4	PCA	[−21.52, 26.56]	12.36 ± 6.24
chest	SNR	4	PCA	[−21.70, 26.61]	12.81 ± 5.55
chest	SE + SNR	4	Average	[−26.96, 31.64]	14.61 ± 8.32
chest	SE	4	Average	[−26.93, 31.57]	14.64 ± 8.42
chest	SNR	4	Average	[−27.42, 31.67]	14.98 ± 8.22

**Figure A2:**
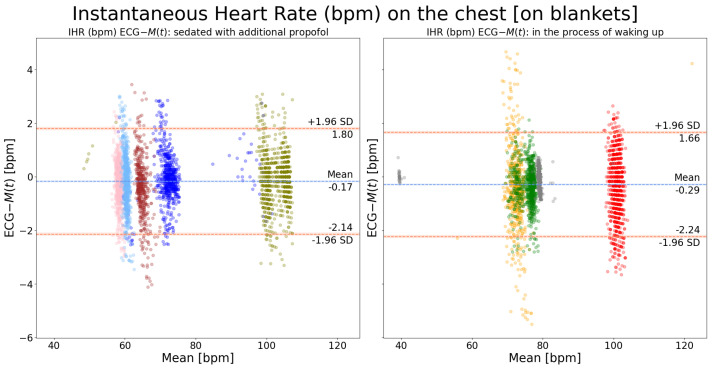
Bland–Altman plot of instantaneous respiration rate on the chest: a comparison between patients with additional propofol administered and patients without. Each color indicates recordings from one patient.

**Figure A3:**
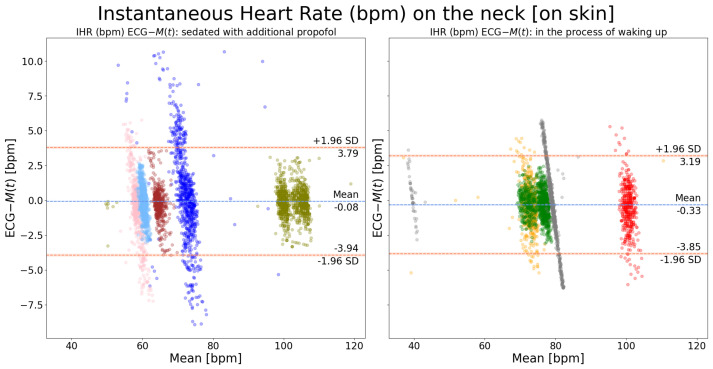
Bland–Altman plot of instantaneous respiration rate on the neck: a comparison between patients with additional propofol administered and patients without. Each color indicates recordings from one patient.

## Data Availability

Data is contained within the article.

## Abbreviations

    The following abbreviations are used in this manuscript:

IHR	Instantaenous heart rate
BPM	Beats per minute
SV	Speckle vibrometry
BMI	Body mass index
SD	Standard deviation
LOA	Limits of agreement
RMSE	Root mean square error
ECG	Electrocardiogram
BCG	Ballistocardiogram
PPG	Photoplethysmogram
SCG	Seismocardiogram
SPG	Speckle plethysmogram
GCG	Gyrocardiogram
LDV	Laser Doppler vibrometry

## Appendix A. Waveform-Based Peak Detection

The pseudo-code of the waveform-based peak detection method is presented in Algorithm A1.

**Algorithm 1** Waveform-based cardiac peak detection for SV	
find_peaks(Signal,Threshold)	▹ the find_peaks function in Python
$P_{W}$	▹ the AO peak location inside W(t)
$W\left(t\right)=\left[w_{1},w_{2},...,w_{A}\right]$	▹ the waveform template with A data points
$\bar{W}\left(t\right)=\frac{W(t)-\min(W(t))}{\max(W(t))-\min(W(t))}-0.5$	▹ normalize W(t)
$M\left(t\right)=\left[m_{1},m_{2},...,m_{N}\right]$	▹ the SV cardiac measurement with N data points
$\bar{M}\left(t\right)=\frac{M(t)-\min(M(t))}{\max(M(t))-\min(M(t))}-0.5$	▹ normalize M(t)
$CC\left(t\right)=\left[cc_{1},cc_{2},...,cc_{N}\right]$	▹ the cross-correlation between $\bar{W}\left(t\right)$ and $\bar{M}\left(t\right)$
$P_{o}\leftarrow \left[\right]$	▹ the initial list of peaks in M(t)
$P_{cc}\leftarrow \left[\right]$	▹ the initial list of peaks in CC(t)
$G_{cc}\leftarrow \left[\right]$	▹ the polarity/sign of peaks in CC(t)

**#(1) locating peaks of the cross-correlation signal** CC(t)	

**while** i≤N **do**	
$W_{i}\left(t\right)=CC\left(t=i:i+A\right)$	
$S_{i}=\frac{3\mu_{W_{i}}}{\sigma_{W_{i}}}$	▹ the skewness of $W_{i}\left(t\right)$
**if** $S_{i}<0$ **then**	
**if** i>1 & $length(P_{cc})>0$ **then**	
**if** $\min\left(W_{i}\left(t\right)\right)+i-P_{cc}\left(i-1\right)<\frac{A}{1.5}$ **then** continue	
**end if**	
**end if**	
$P_{cc}\leftarrow \min\left(W_{i}\left(t\right)\right)+i$	▹ append to the list $P_{cc}$
$G_{cc}\leftarrow -1$	▹ append to the list $G_{cc}$
**else**
**if** i>1 & $length(P_{cc})>0$ **then**	
**if** $\max\left(W_{i}\left(t\right)\right)+i-P_{cc}\left(i-1\right)<\frac{A}{1.5}$ **then** continue	
**end if**	
**end if**	
$P_{cc}\leftarrow \max\left(W_{i}\left(t\right)\right)+i$	
$G_{cc}\leftarrow 1$	
**end if**	
$i=i+\frac{A}{2}$	
**end while**	

**#(2) mapping $P_{cc}$ to their corresponding AO peak locations $P_{o}$ based on waveform comparison with $\bar{W}\left(t\right)$**	

**while** $j\leq length(P_{cc})$ **do**	
**if** j==1 **then**	
$s=P_{cc}\left(j\right)-\frac{A}{2}$	
**else**	
$s=P_{cc}\left(j-1\right)+\frac{P_{cc}\left(j\right)-P_{cc}\left(j-1\right)}{4}$	
**end if**	
**if** $j==length(P_{cc})$ **then**	
$e=P_{cc}\left(j\right)$	
**else**	
$e=P_{cc}\left(j\right)+\frac{0.05}{T}$	▹$\frac{1}{T}$: the sampling frequency (Hz)
**end if**	
**if** s>1 **then**	
$M_{j}\left(t\right)=M\left(t\right)\left(t=s:e\right)$	
**if** $W(P_{W})<0$ **then**	
$Th=percentile^{90}\left(-M_{j}\left(t\right)\right)$	
$P_{j}\left(t\right)=find\_peaks\left(-M_{j}\left(t\right),Th\right)$	
**else**	
$Th=percentile^{90}\left(M_{j}\left(t\right)\right)$	
$P_{j}\left(t\right)=find\_peaks\left(M_{j}\left(t\right),Th\right)$	
**end if**	
**if** $length(P_{j}\left(t\right))==1$ **then**	
$P_{o}\leftarrow P_{j}\left(1\right)$	
**else**	
$c_{x}\left(t\right)=M\left(t\right)\left(t=P_{j}\left(x\right)+s-P_{W}:P_{j}\left(x\right)+s+A-P_{W}\right)$	
$C\left(t\right)=[c_{1}\left(t\right),...,c_{y}\left(t\right)]$	▹$y=length(P_{j}\left(t\right))$
$cc\_list=[cc_{\bar{W}\left(t\right)c_{1}\left(t\right)},...,cc_{\bar{W}\left(t\right)c_{y}\left(t\right)}]$	▹ cc: correlation coefficient
$P_{o}\leftarrow P_{j}\left(argmax\left(cc\_list\right)\right)$	
**end if**	
**end if**	
j=j+1	
**end while**	

## Appendix B. Scatterplots of ECG-Derived IHR and SV-Derived IHR

As can be observed in Figure A1, subjects coded in pink, blue, and grey exhibited an explicit vertical trend on the scatterplot from the neck, but less so from the chest. It is worth noting that the numbers of inter-beat interval (IBI) from both locations are comparable for all subjects despite the peak alignment procedure which selected valid IBI pairs from ECG and M(t). For example, the IBI numbers of the pink subject from the chest and the neck are 543 and 553, respectively. This indicated that such differences in alignment observed from the chest and the neck were not introduced by differing numbers of valid IBI. Instead, it implies that the peak annotation performance from the chest M(t) exceeds that from the neck M(t) for these subjects. Nevertheless, from the chest measurements, it can be observed that the subjects coded in light blue and pink exhibit low heart rate variability (narrowly centered around 60 bpm and 58 bpm, respectively). Both participants underwent aortic valve repair surgery prior to the recording. Before and during the recording, propofol and noradrenaline were administered which presumably reduced the heart rate variability. The heart rate pattern of the subject coded in grey showed two centering points, one around 40 bpm and the other around 80 bpm. The 40 bpm was induced by the patient’s cardiac arrhythmia.

## Appendix C. Performance Comparison between Patients with Additional Propofol Administered and Patients without

Excluding the patient with a pacemaker, there were six patients (color-coded as pink, light blue, brown, blue, purple, and olive green) administered propofol after surgical operations while the others were in the process of waking up during recording. As shown in Figure A2 and Figure A3, the pink, light blue, and purple patients exhibited lower heart rate variability while the brown, blue, and olive green patients still showed a certain degree of heart rate variability despite the potential impact of additionally administered propofol. Among patients without additional propofol administered, the grey patient exhibited two heart rate ranges during recording, one close to 40 bpm and the other close to 80 bpm. The green patient also exhibited a high degree of heart variability as did the propofol-administered patient coded in olive green. The performance of SV does not exhibit a bias concerning different degrees of heart rate variability. Results on patients without additional propofol are comparable to those with.
